# Exercise‐stimulated arterial transit time in calf muscles measured by dynamic contrast‐enhanced magnetic resonance imaging

**DOI:** 10.14814/phy2.13978

**Published:** 2019-01-15

**Authors:** Christopher C. Conlin, Gwenael Layec, Christopher J. Hanrahan, Nan Hu, Michelle T. Mueller, Vivian S. Lee, Jeff L. Zhang

**Affiliations:** ^1^ Department of Radiology and Imaging Sciences University of Utah Salt Lake City Utah; ^2^ School of Public Health and Health Sciences University of Massachusetts Amherst Amherst Massachusetts; ^3^ Division of Biostatistics Department of Internal Medicine University of Utah Salt Lake City Utah; ^4^ Division of Vascular Surgery Department of Internal Medicine University of Utah Salt Lake City Utah; ^5^ Verily Life Sciences Cambridge Massachusetts

**Keywords:** Arterial spin labeling, arterial transit time, calf muscle, perfusion

## Abstract

The primary goal of this study was to evaluate arterial transit time (ATT) in exercise‐stimulated calf muscles as a promising indicator of muscle function. Following plantar flexion, ATT was measured by dynamic contrast‐enhanced (DCE) MRI in young and elderly healthy subjects and patients with peripheral artery disease (PAD). In the young healthy subjects, gastrocnemius ATT decreased significantly (*P* < 0.01) from 4.3 ± 1.5 to 2.4 ± 0.4 sec when exercise load increased from 4 lbs to 16 lbs. For the same load of 4 lbs, gastrocnemius ATT was lower in the elderly healthy subjects (3.2 ± 1.1 sec; *P* = 0.08) and in the PAD patients (2.4 ± 1.2 sec; *P* = 0.02) than in the young healthy subjects. While the sensitivity of the exercise‐stimulated ATT is diagnostically useful, it poses a challenge for arterial spin labeling (ASL), a noncontrast MRI method for measuring muscle perfusion. As a secondary goal of this study, we assessed the impact of ATT on ASL‐measured perfusion with ASL data of multiple post labeling delays (PLDs) acquired from a healthy subject. Perfusion varied substantially with PLD in the activated gastrocnemius, which can be attributed to the ATT variability as verified by a simulation. In conclusion, muscle ATT is sensitive to exercise intensity, and it potentially reflects the functional impact of aging and PAD on calf muscles. For precise measurement of exercise‐stimulated muscle perfusion, it is recommended that ATT be considered when quantifying muscle ASL data.

## Introduction

Peripheral artery disease (PAD) commonly presents as arterial stenosis in the lower extremities (Ouriel 2001). Such stenosis impairs blood supply to the leg muscles and may lead to intermittent claudication (calf weakness or pain during exercise). For the diagnosis of PAD, ankle‐brachial index (ABI) (Ouriel and Zarins 1982) is measured as the ratio of the blood pressure in the ankle of the affected leg to the pressure in the brachial artery. Prior to surgical intervention, angiography using magnetic resonance imaging (MRI) or X‐ray is often applied to determine the location and significance of the stenosis (Carpenter et al. 1994; Hahn et al. 2007; Dewey 2009). Angiography also reveals the growth of collateral arteries, a compensatory mechanism for maintaining tissue perfusion in the affected leg (Greve et al. 2006; Traupe et al. 2013). Researchers have also explored the use of Doppler ultrasound to measure blood velocity and flow in the major peripheral arteries (Scarpello et al. 1980; Radegran 1985). However, none of these clinical measurements reflect how arterial stenosis impacts the delivery of blood to the individual calf muscles, particularly during exercise. Muscle perfusion measured by MRI methods has been extensively explored for its value in assessing calf‐muscle function and performance in PAD patients (Ament et al. 1998; Nygren et al. 2000; Raynaud et al. 2001; Lutz et al. 2004; Boss et al. 2006; Isbell et al. 2007; Brunner et al. 2016; Englund et al. 2016).

Besides muscle perfusion, arterial transit time (ATT) is another promising metric that could provide insight into the delivery of blood to the calf muscles in PAD. In dynamic contrast‐enhanced (DCE) MRI, ATT (often referred to as bolus arrival time in the field of MRI (Cheong et al. 2003; Mehrtash et al. 2016; Paling et al. 2014)) measures the time delay for arterial blood to travel to the capillaries of the tissue from a major upstream artery, such as the popliteal artery, and directly reflects blood transit velocity and/or the route through upstream arteries and arterioles. For PAD patients, we expect that the measured ATT is sensitive to cardiac output, arterial stenosis in the original peripheral arteries, and flow through newly developed collateral arteries. In this sense, ATT is complementary to muscle perfusion, which is more sensitive to local vasodilation during exercise (Morganroth et al. 1975; Vetterlein and Schmidt 1980; Tschakovsky et al. 1996).

The primary aim of this study was to validate ATT as a sensitive metric for assessing the delivery of blood to calf muscles. For this aim, we measured calf‐muscle ATT in young healthy subjects, stimulated by plantar‐flexion exercise of different intensities. As a pilot investigation, we also compared the ATT estimates from these young healthy subjects to those from elderly control subjects and PAD patients.

Arterial spin labeling (ASL) is an MRI technique widely used to measure skeletal muscle perfusion (Raynaud et al. 2001; Boss et al. 2006; Andreisek et al. 2009; Wray et al. 2009; Pollak et al. 2012; Englund et al. 2016; Chen and Wright 2017). The sensitivity of ATT to exercise stimulation and the wide range of ATT values observed in exercise‐stimulated calf muscles prompted the second aim of this study: to assess the impact of ATT on muscle perfusion measurement with ASL. The interaction between ATT and ASL perfusion has been widely investigated in the brain, and multiple innovative approaches have been designed to account for it (Thomas et al. 2006; Qiu et al. 2010; Hirschler et al. 2018). One such method measures both ATT and perfusion from ASL data, and has been applied to calf muscles during ischemic hyperemia (cuffing) (Wu et al. 2008). For skeletal muscles, however, the exercise stimulation used in the proposed study better reflects natural muscle activation, and also induces more dramatic changes in ATT (Yu et al. 2005). For this aim, we acquired ASL data from the calf muscles of a healthy subject and demonstrated the impact of ATT by comparing perfusion estimates obtained using different post labeling delays (PLDs). A simulation of the muscle ASL signal was also performed to further interpret the results.

## Materials and Methods

This study was approved by the local institutional review board. Nineteen subjects in total were recruited for this study, and each subject provided written informed consent before undergoing any experimental procedures. For the primary aim of this study, DCE MRI was used to measure ATT from eight young healthy subjects (age: 26.0 ± 4.6 years; 6 male; BMI: 24.4 ± 3.1). For the pilot investigation, the same DCE MRI protocol was performed for five elderly control subjects (age: 62.6 ± 4.3 years; 4 male; BMI: 28.4 ± 3.5) and five patients with PAD (age: 62.2 ± 4.8 years; 3 male; BMI: 26.4 ± 5.8; ABI: 0.56–0.84). Of the five elderly control subjects, none had history or symptom of peripheral vascular disease such as leg pain or intermittent claudication, cardiovascular disease, or diabetes. Of the five PAD patients, three were diagnosed by computer‐tomography angiographic data, showing the presence of significant peripheral artery stenosis, and the other two had intermittent claudication and ABI of less than 0.9. According to our exclusion criteria, the patients did not have chronic pulmonary diseases or joint problems that might impede their ability to perform plantar‐flexion exercise, or impaired renal function (estimated GFR below 30 mL/min/1.73 m^2^). Of the five PAD patients, two had type‐II diabetes and five had hypertension. For the second aim of this study, ASL MRI was repeated with multiple PLDs in a healthy volunteer (age: 25 years; male; BMI 23.8). All MRI measurements were performed immediately following plantar‐flexion exercise stimulation.

### DCE MRI to measure exercise‐stimulated ATT

All MRI scans were performed on a 3T clinical scanner (TimTrio or Prisma fit; Siemens) equipped with an MRI‐compatible exercise apparatus. The apparatus allowed the subject to perform unilateral plantar flexion with adjustable load while lying supine in the scanner bore. A flexible 4‐channel receiver coil was wrapped around the calf of the exercising leg. The primary protocol for this study involved plantar flexion with a load of 4 lbs for 3 min at a frequency of 1 Hz. To ensure the dynamic plantar flexion to be completed, a metronome was used to help the subject synchronize the exercise to the desired frequency, and a study‐team member was present by the MR scanner to supervise the exercise. Five seconds before the exercise ended, 0.05 mmol/kg of gadoteridol (Prohance; Bracco) tracer was injected intravenously at a rate of 5 mL/sec, followed by a 20 mL saline flush at the same rate. Immediately following exercise, dynamic MR imaging of the calf was performed with a saturation‐recovery prepared FLASH sequence: repetition time (TR) 527 msec, echo time (TE) 1.42 msec, time delay 307 msec, flip angle 15°, slice thickness 10 mm, matrix 128 × 128, field of view (FOV) 160 × 160 mm. An axial slice covering the maximal cross‐sectional area of the calf was imaged. Imaging continued for 4 minutes with a temporal resolution of 1 image per second. To quantify tracer concentration from the dynamic images, we acquired additional proton‐density‐weighted images using the same sequence and parameter values, but with a long TR of 4000 msec. Besides the exercise load of 4 lbs, for the eight young healthy subjects the protocol was repeated two additional times, once with an 8‐lb load and once with a 16‐lb load.

The acquired MRI data was exported to a personal computer for post processing with custom programs written in MATLAB (The MathWorks, Inc). Each dynamic image was first converted to a map of tracer concentration based on the proton‐density‐weighted images using a voxel‐wise approach (Vivier et al. 2011). An arterial input function (AIF) was sampled from the largest artery visible in the imaging slice, typically the peroneal artery. An impulse retention function (IRF) approach (St Lawrence KS 1998; Koh et al. 2011) was used to estimate the time delay of contrast enhancement in each muscle voxel relative to the enhancement in the artery (i.e., AIF), or arterial transit time (ATT). By computing ATT for each voxel in the image slice, we obtained a map of ATT. Our analysis in this study focused on four muscle groups: medial gastrocnemius (MG), lateral gastrocnemius (LG), soleus (S), and tibialis anterior (AT), for each of which we manually defined regions of interest (ROIs) to compute mean and standard deviation of the ATT values.

With the collected data, we assessed the potential sensitivity of muscle ATT to two factors: 1) exercise intensity: plantar flexion was performed in the young healthy subjects using three different loads: 4, 8, and 16 lbs; and 2) muscle activation, which should be different for the four muscle groups (MG, LG, S, and AT) during plantar flexion. A linear mixed‐effects statistical model (Mclean et al. 1991) was used to test if changes in ATT with exercise intensity were significant. The Kruskal–Wallis test (Kruskal and Wallis 1952) was used to determine if there was any significant difference in muscle activation among the four muscle groups, and if there was, pairwise *t*‐tests were used to identify which pairs differed from each other. Finally, we compared the ATT between the three pilot groups (young healthy, elderly healthy, and PAD patients), using analysis of variance (ANOVA), and if any significant difference was detected, two sample *t*‐tests were used to identify which two groups differed from each other. For all comparisons, a *P* < 0.05 was regarded as significant.

### Exercise‐stimulated calf‐muscle perfusion from ASL

This experiment was designed to assess the impact of muscle ATT on ASL‐estimated perfusion. In practice, it is difficult to induce a precise change in muscle ATT. Instead, we can vary the PLD of the ASL acquisition, which is the time between arterial labeling and the actual image readout. Increasing PLD has essentially the same effect as decreasing ATT on the acquired ASL signals (Wong 2005; Petersen et al. 2006). With the same exercise setup as in DCE MRI, a healthy volunteer (age: 25 years; male) performed plantar‐flexion exercise with a load of 16 lbs for 3 min at a frequency of 1 Hz. Immediately after exercise, ASL MRI of the calf was performed using a flow‐sensitive alternating inversion‐recovery (FAIR) labeling approach (Kim and Tsekos 1997). In FAIR labeling, two images are acquired following different inversion pulses: one after a spatially nonselective inversion and the other after a slice‐selective inversion, both centered on the imaging slice. A “quantitative imaging of perfusion using a single subtraction” (QUIPSS) II (Wong et al. 1998) saturation pulse was applied during the labeling, so that a well‐defined bolus of labeled blood was created with a temporal width of 700 msec. Prior to image readout, a PLD was included to allow the labeled blood to enter the imaging slice. The image was then acquired using a turbo gradient spin echo (TGSE) pulse sequence: TR 6 sec, TE 19.24 msec, echo train length 21, matrix size 128 × 128, FOV 169 × 169 mm^2^, slice thickness 3 mm. The imaging slice was oriented axially, at the level of maximal cross‐sectional area of the calf. The above exercise and imaging procedure were repeated with four different PLD values: 800, 1200, 1600, and 2000 msec. A 15‐min rest was included between the repetitions to allow the subject's calf to recover to baseline physiological condition.

From each of the four ASL image sets, we obtained a perfusion map using the recommended single‐PLD formula from the ISMRM perfusion study group (Alsop et al. 2015). For the medial gastrocnemius (MG), soleus (S), and tibialis anterior (AT), ROIs were manually defined to obtain average perfusion estimates at the four different PLD values. The lateral gastrocnemius (LG) was not considered due to its small area in the field of view. For each muscle group, the perfusion estimates were plotted against the PLD values to demonstrate the potential dependence of ASL‐measured perfusion on PLD. With ASL data acquired at multiple PLD values, we had the opportunity to quantify voxel‐wise muscle perfusion using a tracer‐kinetic approach proposed by Buxton et al. (Buxton et al. 1998), a method that considers the entire bolus of labeled blood and thus does not depend on PLD or ATT. The multi‐PLD and single‐PLD perfusion maps were displayed to appreciate the impact of ATT variability on the estimation of voxel‐wise perfusion.

To help interpret the above human ASL results, we simulated muscle ASL signals (Buxton et al. 1998) for two levels of muscle activation status. The high‐activation muscle was characterized by high perfusion (300 mL/min/100 g) and low ATT (50 msec), while the low one by lower perfusion (150 mL/min/100 g) and higher ATT (500 msec). These values were chosen based on the multi‐PLD perfusion results described above. Other simulation parameters were the same for the two muscle activation levels: T_1_ of blood 1650 msec, T_1_ of muscle 1400 msec, labeling duration 700 msec, blood‐tissue partition coefficient 0.9 mL/g, and PLD from 0 to 2000 msec (with an interval of 1 msec). For the simulated signals at each PLD and activation level, a perfusion estimate was calculated using the ISMRM perfusion study group formula (Alsop et al. 2015). For both the activation levels, the simulated perfusion was plotted against all PLD values.

## Results

### Sensitivity of ATT to exercise load and subject group

**Figure 1:**
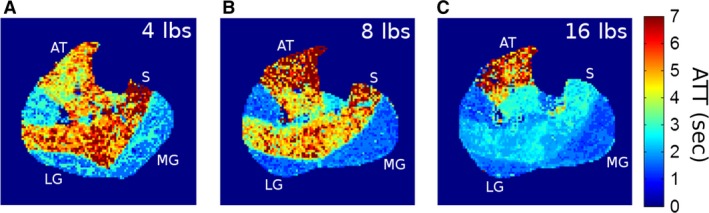
Representative ATT maps from a young healthy subject after plantar flexion of different loads: (A) 4 lbs, (B) 8 lbs, and (C) 16 lbs. ATT values varied markedly between different muscle groups and between different loads. Low ATT value corresponds to high velocity of arterial blood. MG, medial gastrocnemius; LG, lateral gastrocnemius; S, soleus; AT, tibialis anterior.

**Figure 2:**
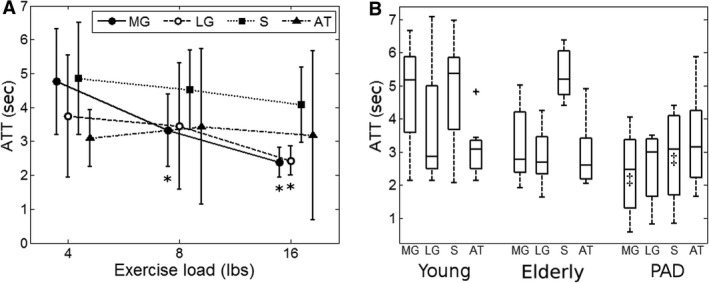
Calf‐muscle ATT measured by DCE MRI during recovery after plantar flexion. (A) Error‐bar plot (mean ± standard deviation) for ATT at different loads for the young healthy subjects. (B) Box‐and‐whisker plot for ATT of the different subject groups at the same load of 4 lbs (outliers marked with a “+”). Significant differences in ATT, compared to the values from young healthy subjects at the 4‐lb exercise load, are indicated by * for the comparison between exercise loads (A) and by & for the comparison between subject groups (B). A *P *< 0.05 is regarded as significant. MG: medial gastrocnemius; LG, lateral gastrocnemius; S, soleus; AT, tibialis anterior.

Figure 1 shows representative maps of ATT stimulated by plantar flexion of different intensity for a young healthy subject. The ATT values for all the eight subjects are summarized in Table 1. For the entire gastrocnemius (MG and LG), ATT decreased from 4.3 ± 1.5 sec at 4 lbs to 3.3 ± 1.2 sec at 8 lbs, and further to 2.4 ± 0.4 sec at 16 lbs (*P* < 0.01). The two gastrocnemius subgroups followed comparable decreasing trends (MG: *P* < 0.01, LG: *P* = 0.03). Soleus ATT decreased slightly with increasing load (*P* = 0.22). The tibialis anterior was not supposed to be activated by plantar flexion, and its ATT did not decrease with load (*P* = 0.53). Figure 2A shows the general trend of ATT values for the different muscle groups and exercise loads.

**Table 1:** Calf‐muscle ATT values (unit: sec) measured by DCE MRI during recovery after plantar flexion

ATT (sec)	G	MG	LG	S	AT
Young – 4 lbs	4.3 ± 1.5	4.8 ± 1.6	3.7 ± 1.8	4.9 ± 1.6	3.1 ± 0.8
Young – 8 lbs	3.3 ± 1.2*	3.3 ± 1.1*	3.4 ± 1.9	4.5 ± 1.2	3.4 ± 2.3
Young – 16 lbs	2.4 ± 0.4*	2.4 ± 0.4*	2.4 ± 0.4*	4.1 ± 1.1	3.2 ± 2.5
Elderly – 4 lbs	3.2 ± 1.1	3.2 ± 1.2	2.9 ± 1.0	5.4 ± 0.8	2.9 ± 1.2
PAD – 4 lbs	2.4 ± 1.2‡	2.4 ± 1.4‡	2.5 ± 1.1	2.9 ± 1.5‡	3.4 ± 1.6

The values are mean ± standard deviation across different subjects in each group (eight young healthy subjects, five elderly control subjects, and five PAD patients). Significant differences in ATT, compared to the values from young healthy subjects at the 4‐lb exercise load, are indicated by * for the comparison between exercise loads and by & for the comparison between subject groups. A *P*‐value of less than 0.05 is regarded as significant.

MG, medial gastrocnemius; LG, lateral gastrocnemius; S, soleus; AT, tibialis anterior; G, the entire gastrocnemius (i.e., MG and LG).

Arterial transit time values were also compared between the different groups of subjects (young, elderly, and PAD) following planter flexion of the same intensity of 4‐lb load. Compared to the young healthy subjects (4.3 ± 1.5 sec), gastrocnemius ATT was significantly lower in the PAD patients (2.4 ± 1.2 sec, *P* = 0.02), and lower but not significantly in the elderly control subjects (3.2 ± 1.1 sec, *P* = 0.08). Similar results were observed for the soleus: young healthy subjects 4.9 ± 1.6 sec, elderly healthy subjects 5.4 ± 0.8 sec (*P* = 0.54), and PAD patients 2.9 ± 1.5 sec (*P* = 0.04). No significant difference was detected between tibialis anterior ATT of the three subject groups (*P* = 0.85). A graphical comparison of ATT between the different subject groups is shown using box‐and‐whisker plots in Figure 2B.

### Dependence of ASL perfusion estimates on PLD/ATT

**Figure 3:**
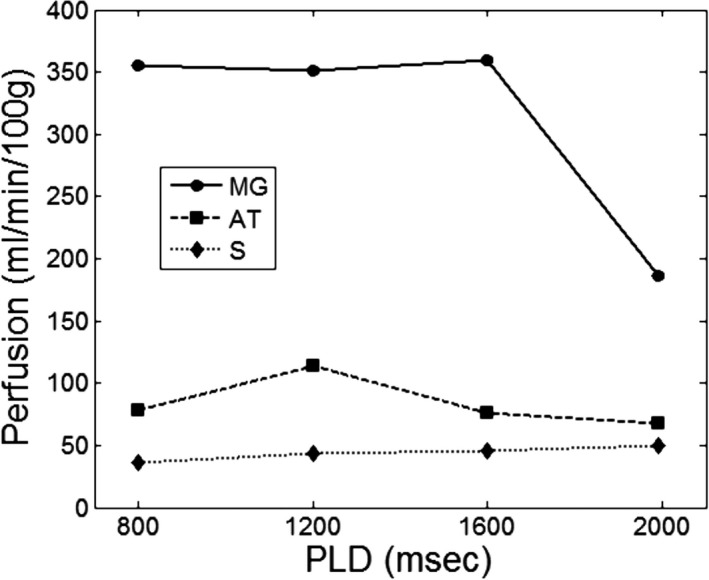
ASL perfusion estimates from the calf of a healthy volunteer, calculated at four different PLDs using a single‐PLD formula. Average perfusion values are shown for three calf muscle groups: medial gastrocnemius (MG), tibialis anterior (AT), and soleus (S).

**Figure 4:**
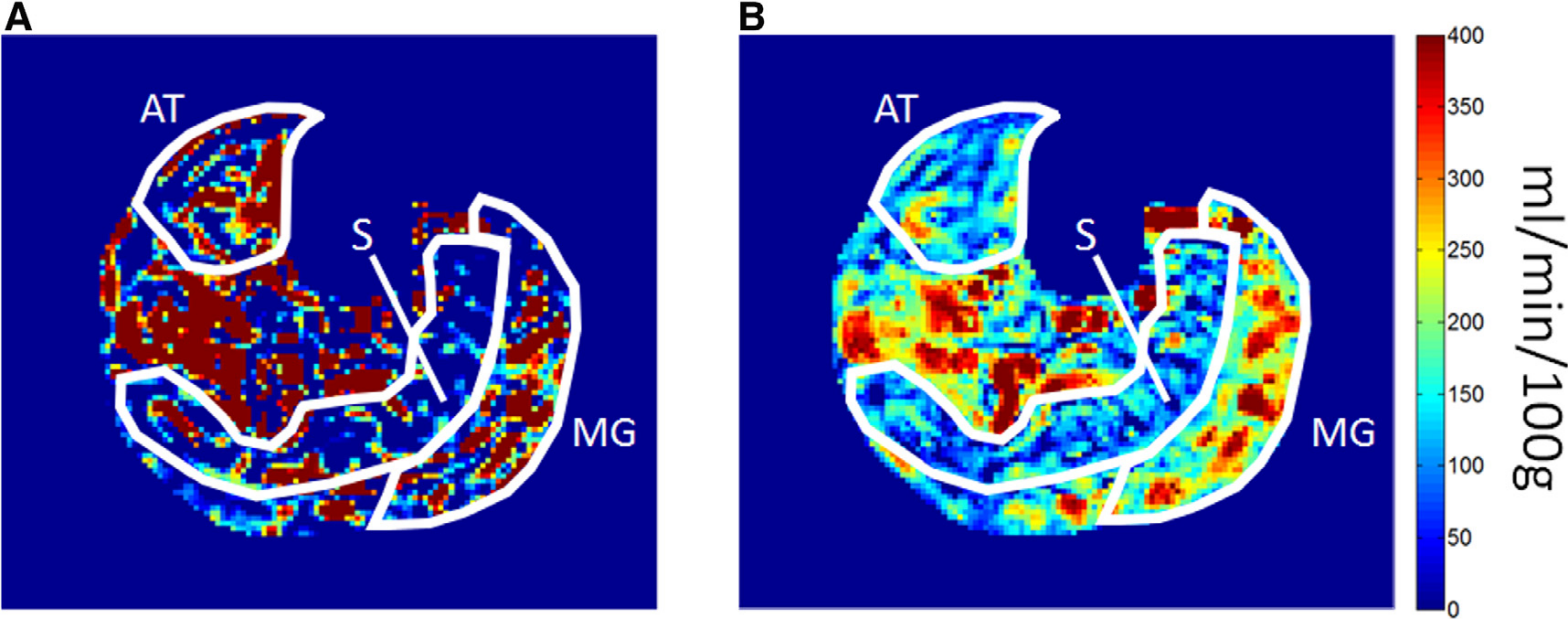
ASL perfusion maps computed using a single PLD (A) and multiple PLDs (B). Medial gastrocnemius (G), soleus (S), and tibialis anterior (AT) muscle groups are outlined in white. The single‐PLD map was calculated from the ASL signal at PLD 1200 msec, using the ISMRM perfusion study group formula. The multi‐PLD map was computed by fitting the ASL signals at four different PLDs (800, 1200, 1600, 2000 msec) to a tracer‐kinetic model of the ASL signal.

Figure 3 shows exercise‐stimulated perfusion estimates in a healthy volunteer's MG, AT, and S, as measured by ASL acquisitions using different PLD values. MG perfusion was stable at 355 ± 4 mL/min/100 g for PLD of 800–1600 msec, and decreased to only 187 mL/min/100 g at PLD of 2000 msec. AT perfusion was much lower than MG perfusion, averaging 84 ± 20 mL/min/100 g over all PLDs. Soleus perfusion was lower than in either the MG or AT, averaging 44 ± 6 mL/min/100 g over all PLDs. There was a trend of increasing soleus perfusion with increasing PLD: from 36 mL/min/100 g at PLD 800 msec to 50 mL/min/100 g at PLD 2000 msec, but it was not statistically significant. To appreciate the impact of ATT variability on ASL perfusion quantification on a voxel‐wise basis, a perfusion map estimated from ASL data of PLD 1200 msec is shown in Figure 4A. For a large proportion of voxels in the map, perfusion values were either lower than 0 or higher than 400 mL/min/100 g. In contrast, quantification of the ASL data using all four PLDs resulted in a perfusion map (Fig. 4B) with much less noise and obvious hyperperfusion in the gastrocnemius.

**Figure 5:**
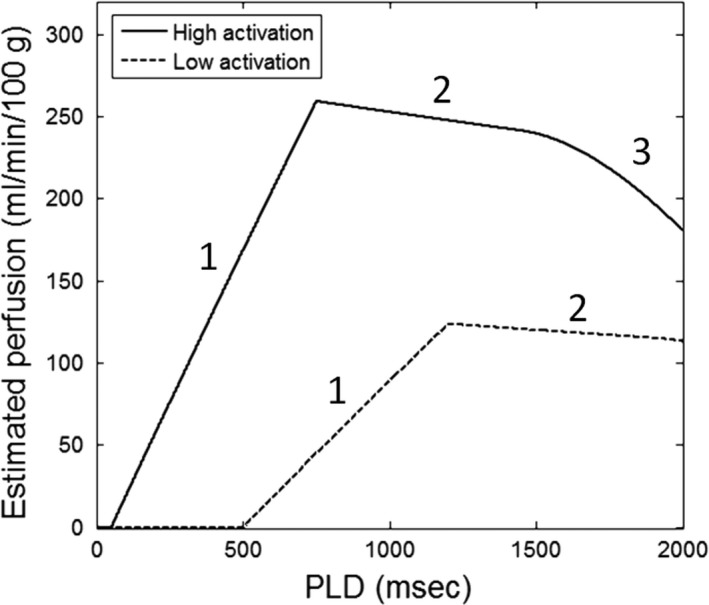
Simulated ASL perfusion estimates, calculated over a range of PLDs using a widely used single‐PLD formula. Muscles with high and low levels of activation by plantar‐flexion exercise were simulated. The simulation used a labeling duration (bolus width) of 700 msec, and an ATT of 50 msec for the muscle with high activation and 500 msec for the muscle with low activation. The labeled intervals of the perfusion estimate versus PLD curve correspond to different tracer‐kinetic phases of the labeled blood: (1) Labeled blood is still flowing into the muscle, (2) The entire bolus of labeled blood has been delivered to the muscle, and (3) Labeled blood is washing out of the muscle.

The relationship between PLD and estimated perfusion can be further demonstrated by the ASL simulation of two muscle activation levels in Figure 5. The curves show distinct intervals that corresponds to different tracer‐kinetic phases of the ASL signal: inflow, complete‐bolus delivery, and then washout. Over the timescale of this simulation from 0 to 2000 msec, stable perfusion estimates can be derived from PLDs in the range of 700–1500 msec for the high‐activation muscle. For the low‐activation muscle, due to its long ATT, stable perfusion estimates can only be derived from PLDs longer than 1200 msec.

## Discussion

The primary aim of this study was to test the feasibility of assessing calf‐muscle function through measurement of exercise‐stimulated ATT using DCE MRI. We found that in young healthy subjects after exercise stimulation by plantar flexion, ATT of the gastrocnemius progressively decreased as exercise intensity increased, and that this progressive change was not observed in the soleus or the anterior tibialis, muscle groups that do not support straight‐leg plantar flexion. The ATT changes in the gastrocnemius presumably reflect exercise‐induced changes in cardiac output (Meyer et al. 1993) and muscle vascular conductance (Villar and Hughson 2017). For the same low‐intensity exercise, gastrocnemius ATT was significantly lower in the elderly control subjects and the PAD patients than in the young healthy subjects. Lower ATT values in the elderly subjects could be due to age‐related adaptation of endothelial function (Padilla et al. 2011), and for some PAD patients, elevated cardiac output to compensate for mitochondrial dysfunction in the muscle (Regensteiner et al. 1993; Ferguson et al. 2001; Pipinos et al. 2003; Brass et al. 2004).

Magnetic resonance imaging as a tool for assessing skeletal muscle function has been mostly used to measure muscle perfusion (Frank et al. 1999; Isbell et al. 2007) and oxygenation (Ledermann et al. 2006; Muller et al. 2016; Suo et al. 2018). Exercise‐stimulated ATT as measured by the proposed method would be another potentially useful parameter for assessing muscle function, particularly for PAD patients where the delivery of blood to particular calf muscles is often impeded by peripheral artery stenoses and reduced capillary density. One convenient feature of muscle ATT is that it can be quantified from the same DCE MRI dataset as muscle perfusion. While our proposed study is the first evaluation of ATT in exercise‐stimulated calf muscles, the potential value of ATT has already been explored in other organs. In functional MRI studies of the brain (Gonzalez‐At et al. 2000; Yang et al. 2000), ATT was found to decrease in activated regions of the brain as result of increased blood flow velocity to deliver additional oxygen to the activated tissue (Aaslid 1987; Conrad and Klingelhofer 1989), similar to the decrease in gastrocnemius ATT after stimulation by plantar‐flexion exercise in our study. Other organs in which ATT has been studied include the liver (Sugimoto et al. 2002; Hohmann et al. 2012) and myocardium (Wang et al. 2010; Pelgrim et al. 2016).

The wide range of ATT observed in the calf, stemming from different activation responses of the muscles to plantar flexion, would present a significant challenge for quantifying calf‐muscle perfusion using ASL. As demonstrated by our simulation of different muscle groups, conventional single‐PLD perfusion estimates varied substantially with PLD selection in the different tracer‐kinetic phases of the labeled blood (Fig. 5). Accurate perfusion estimation can only be achieved when the entire bolus of labeled blood is within the muscle tissue (phase 2 in Fig. 5), that is, with appropriate PLD selection relative to a muscle's ATT value. However, as we observed in this study, ATT differs between muscle groups and is sensitive to exercise intensity. A PLD value appropriate for a muscle with long ATT may be too late for a muscle with short ATT, such that the ASL signal is measured during the wash‐out phase, thereby underestimating muscle perfusion (e.g., the estimated MG perfusion at PLD of 2000 msec in Figure 3). Hence, it could be impossible to determine a single PLD value that accommodates the wide range of ATT values present in the calf, let alone for comparison between different exercise intensities.

To overcome the confounding effect of ATT in ASL perfusion estimation, an ASL‐acquisition protocol with multiple PLD values may be considered (Thomas et al. 2006; Kim et al. 2017). Using a tracer‐kinetic model such as proposed by Buxton et al. to fit the multi‐PLD data, a more robust estimation of muscle perfusion can be achieved, as illustrated by the perfusion map comparison in Figure 4. At present, however, the multi‐PLD approach is impractical for exercise‐stimulated muscles, because between the multiple exercise stimulations, a relatively long period is needed to allow the muscles to return to a resting physiological state. One promising technique for accelerating the multi‐PLD protocol is via time‐encoded continuous ASL (te‐CASL) (Thomas et al. 2006; Dai et al. 2013; Hirschler et al. 2018), in which the labeling period is divided into multiple blocks that alternate between inversion and noninversion states. The individual blocks are essentially different PLDs, but within a single acquisition. The different blocks can be decoded in post processing, enabling the calculation of perfusion images from multiple PLDs without needing to repeat the acquisition at each PLD. te‐CASL was tested in skeletal muscles with postischemic hyperemia (Wu et al. 2008), and its feasibility for exercise stimulation remains to be tested. We believe that further development of noncontrast imaging such as ASL for assessing calf muscles is worth pursuing for multiple reasons. First, renal dysfunction is common in patients with PAD (DeLoach and Mohler 2007). The risk of nephrogenic systemic fibrosis from gadolinium‐based tracers may preclude the use of DCE MRI in these patients. Second, while both DCE and ASL methods can measure ATT in muscle, ASL‐measured ATT has unique value for assessing muscle arterioles and capillaries only (due to the shorter transit distance from the labeling region to the imaging slice), and not larger arteries as in DCE MRI.

This study has a few limitations. First, while age was comparable between the elderly healthy controls and the PAD patients, we did not consider the potential impact from other factors such as cardiovascular disease, diabetes, or physical activity level. In future study with large population, we will consider these factors in data analysis and interpretation. Second, we did not control for factors such as dietary, medication, and measurement time of day, which may vary the cardiovascular response to exercise. In future work, we will perform experiments to evaluate the impact of each individual factor to our measured ATT. Third, the measured ATT value depends on the arterial location where AIF is sampled, and this should be considered when comparing ATT values from different subjects or studies.

In conclusion, the proposed exercise‐stimulated ATT varies substantially between different muscle groups, is sensitive to exercise intensity, and can potentially assess the functional impact of aging and PAD. In addition to muscle perfusion estimated from the same DCE data, the measurement of calf‐muscle ATT provides a more complete picture of the muscle's hemodynamic state for patients with PAD. The large variability of calf‐muscle ATT also motivates continued effort toward improving muscle perfusion estimation with ASL.

## Conflict of Interest

None declared.
